# Termination of prehospital resuscitative efforts: a study of documentation on ethical considerations at the scene

**DOI:** 10.1186/s13049-017-0381-1

**Published:** 2017-03-31

**Authors:** Søren Mikkelsen, Caroline Schaffalitzky de Muckadell, Lars Grassmé Binderup, Hans Morten Lossius, Palle Toft, Annmarie Touborg Lassen

**Affiliations:** 1 Mobile Emergency Care Unit, Department of Anaesthesiology and Intensive Care Medicine V, Odense University Hospital, Odense, Denmark; 2 Department of Clinical Research, University of Southern Denmark, Odense, Denmark; 3 Philosophy, Department for the Study of Culture, University of Southern Denmark, Odense, Denmark; 4 Field of Prehospital Critical Care, Network for Medical Sciences, University of Stavanger, Kjell Arholmsgate 41, NO-4036, Stavanger, Norway; 5 Norwegian Air Ambulance Foundation, Holterveien 24, NO-1441, Drøbak, Norway; 6 Department of Anaesthesiology and Intensive Care Medicine V, Odense University Hospital, Odense, Denmark; 7 Department of Emergency Medicine, Odense University Hospital, Odense, Denmark

## Abstract

**Background:**

Discussions on ethical aspects of life-and-death decisions within the hospital are often made in plenary. The prehospital physician, however, may be faced with ethical dilemmas in life-and-death decisions when time-critical decisions to initiate or refrain from resuscitative efforts need to be taken without the possibility to discuss matters with colleagues. Little is known whether these considerations regarding ethical issues in crucial life-and-death decisions are documented prehospitally. This is a review of the ethical considerations documented in the prehospital medical records of patients in a Danish prehospital setting for whom the decision to resuscitate or not was made at the scene.

**Methods:**

The study is based on discharge summaries of all patients subjected to crucial life-and-death decisions by the Mobile Emergency Care Unit in Odense in the years 2010 to 2014. The medical records with possible documentation of ethical issues were independently reviewed by two philosophers in order to identify explicit ethical or philosophical considerations pertaining to the decision to resuscitate or not.

**Results:**

In total, 1275 patients were either declared dead at the scene without exhibiting layman’s reliable signs of death or admitted to hospital following resuscitation.

In a total of 62 patients, 85 specific ethical issues related to resuscitation were documented. The expressions of the ethical considerations were generally vague or unclear and almost exclusively concerned the interests of the patient and not the relatives. In the vast majority of cases where an ethical content was identified, the ethical considerations led to a decision to terminate treatment.

**Conclusions:**

A strengthened practice of documenting ethical considerations in prehospital life-and-death decision-making in the patient’s medical records is required. We suggest that a template be implemented in the prehospital medical records describing the basis for any ethical decisions. This template should contain information regarding the persons involved in the deliberations and notes on ethical considerations. The documentation should include considerations concerning the patient’s end-of-life wishes, the estimations of the quality of life before and after the incident, and a summary of other ethical concerns taken into account, such as the integrity of the patient and frame of mind of relatives.

## Background

In Denmark, as in most other countries, physicians are responsible for the act of declaring a patient dead in the prehospital field [1]. By Executive Order issued by the Danish Ministry of Health, the emergency medical technician (EMT) or paramedic (PM) is obliged to initiate resuscitative efforts in all lifeless patients; until declared dead by a physician, all lifeless patients are legally regarded not as dead but rather as patients with cardiac arrest. The sole exceptions are cases in which the EMT documents findings of cadaverositas (signs of decay) or lesions incompatible with life. In these cases, the EMT is allowed not to attempt to resuscitate the patient [2]. In contrast, the physician is entrusted to declare a patient dead on the basis of arrested circulation alone. The decisions whether resuscitative attempts should be initiated or whether a patient in cardiac arrest should be considered dead are complex and multifaceted. The issue of medical futility includes several considerations: evaluation of the probability of benefit for the patient, analysis of the potential harm that the treatment may cause, the costs for patient or society, and, not least, the wishes of the patient. As such, the term medical futility may be approached in different ways. One approach to this issue is that the autonomy of the patient is of paramount importance when deciding whether to initiate, continue or discontinue treatment [3]. Recently, this perception has gained ground, and a shift has been recommended towards a balanced patient-centered approach with greater emphasis on patient autonomy [4]. This approach, however, requires that the wishes and expectations of the patient are known beforehand. If not, the traditional medical-centered approach with an emphasis on beneficence and non-maleficence, which are almost entirely the prerogative of the physician, may be used. Within the hospital, discussions about whether natural death should be allowed or not usually take place between several physicians (as in conferences in the Intensive Care Unit or within a medical emergency team). Prehospitally, however, in the absence of information regarding the wishes and expectations of the patient, the decision to allow natural death must be made by the single physician present (i.e., the emergency care physician). Accordingly, not only strictly medical, but also ethical and similar philosophical considerations must be conducted on the spot without the support and advice from fellow physicians involved in treating the patient. To what extent these considerations are performed and documented in the medical records of the patients is not known. We have previously investigated the causes and consequences of termination of prehospital resuscitation by anaesthesiologists [5]. While performing that study, we were intrigued by what appeared to be a notable scarcity of documented ethical considerations in the patients’ medical records.

The aims of this study are thus to investigate to what extent ethical considerations are documented in discharge summaries in cases of life-and-death decisions made by emergency care anaesthesiologists in a Danish prehospital setting. Furthermore, the study aims to describe the nature of such considerations and seeks to argue for the establishment of recommendations for documentation of ethical considerations in discharge summaries.

## Methods

### Description of study context

The mobile emergency care unit (MECU) in Odense operates as a part of a three-tiered system in which the MECU supplements an ordinary ambulance manned with two EMTs or an ambulance assisted by a PM. The MECU in Odense consists of one rapid-response car, operating all year round, manned with a specialist in anaesthesiology and an EMT.

The MECU is dispatched either by the Dispatch Centre on the basis of the information given by the caller or by request from the EMTs on the primary ambulance [6].

Conforming to Danish law, in all cases where the MECU has an encounter with a patient, the treatment is documented by the MECU physician in the patient’s medical records in the form of a discharge summary. This discharge summary is entered into the patient’s in-hospital medical records and forwarded to the patient’s general practitioner [7]. Furthermore, patient characteristics (including the patient’s unique civil personal registration number as identification) are entered into a MECU registry containing information on all patients treated by the MECU [8]. The physician responsible for the treatment registers both the treatment administered by the MECU and the immediate patient outcome. This assessment of outcome is graded within several categories ranging from “Lifesaving effort” to “Patient declared dead” with or without reliable signs of death.

### Study design

This study is a retrospective study based on the documentation from the MECU registry and the MECU discharge summaries pertaining to each ambulance run performed by the Mobile Emergency Care Unit in Odense Denmark to lifeless patients who did not display reliable signs of death. All patients in whom the decision to allow for natural death was made were included. The study period was January 1st 2010 to December 31st 2014.

### Data analysis

All medical records of the patients included in the study were manually reviewed. All records with possible documentation of ethical issues concerning withholding or withdrawal of resuscitative therapy or initiation of therapy perceived as futile by the attending anaesthesiologists were extracted from the material. These medical records were subsequently reviewed by two philosophers, authors LGB and CSM, and subjected to independent analyses of possible philosophical content in order to provide descriptions and categorization of central ethical content found in the records. Preceding the analyses of the records, a very broad and stipulative definition of “ethical considerations or content” was specified and included considerations regarding the interest of the patient, the interest of the relatives, and other concerns with ethical content. Furthermore, we sought to establish whether any considerations were documented that were not purely medical but pertained to matters of value, prioritisation of conflicting interests, goals of the patient and relatives, and the quality and meaning of the patient’s life.

Following the independent analyses, the results were compared and mutually agreed upon by the philosophers.

### Descriptive analysis and statistical methods

Quantitative data are presented as proportions, median and quartiles or range (where appropriate). Proportions are presented with 95% confidence intervals (CI) based on a binomial distribution. All quantitative data were analysed using non-parametric statistics (Chi-square or Kruskal-Wallis). Differences were considered significant when *p* < 0.05.

All statistical calculations were performed using STATA 14.1 (StataCorp, Texas, USA).

### Legislative approval of the study

The study was approved by the Danish Data Protection Agency (2008-56-0035/15/34069) and the Danish Health and Medicines Authority (3-3013-682/1/). According to Danish legislation, approval from Ethics Boards is not required in registry based observational studies.

## Results

During the study period of five years, a total of 17,035 patients were treated by the MECU. In 1275 patients, the crucial decision to resuscitate or to allow natural death was made by the MECU physician at the scene. In 642 of these patients, resuscitative efforts were initiated at the scene. In 633 patients, natural death was allowed without any resuscitative efforts being initiated.

Demographic data, data regarding the presence of bystanders, the place of the incident, and any illnesses brought to the awareness of the MECU physician at the time of decision making are seen in Table 1.

**Table 1:** Documentation of parameters potentially influencing the MECU-anesthesiologist’s decision to resuscitate or not to resuscitate

	Resuscitative efforts initiated	Dead without treatment	Test	Level of significance
n	642	633		
Sex F/M	212/430	288/345	Chi^2^	<0.0001
Age (Median, Quartiles)	68 years (57, 78)	77 years (65, 85)	Kruskal-Wallis	<0.0001
Presence of bystanders			Chi^2^ (2 × 6 table)	<0.0001
None (n (% 95% CI))	47 (7.3% (5.4–9.1%))	53 (8.4% (6.3–10.8%))		
Next of kin (n (% 95% CI))	277 (43.1% (39.3–47.1%))	252 (39.8% (36.0–43.7%))		
Caregivers (n (% 95% CI))	70 (10.9% (8.6–13.6%))	210 (33.2% (29.5–37.0%))		
Health care workers (n (% 95% CI))	75 (11.7% (9.3–14.4%))	23 (3.6% (2.3–5.4%))		
Others (n (% 95% CI))	133 (20.7% (17.6–24.1%))	51 (8.1% (6.1–10.5%))		
No information available (n (% 95% CI))	40 (6.2% (4.5–8.4%))	44 (7.0% (5.1–9.2%))		
Place of incident			Chi^2^ (2 × 5 table)	<0.0001
Home (n (% 95% CI))	409 (63.7% (59.9–67.4%))	421 (66.5% (62.7–70.2%))		
Nursing home (n (% 95% CI))	49 (7.6% (5.7–10.0%))	142 (22.4% (19.2–25.9%))		
Public place (n (% 95% CI))	144 (22.4% (19.3–25.9%))	44 (7.0% (5.1–9.2%))		
Other (n (% 95% CI))	20 (3.1% (1.9–4.8%))	11 (1.7% (0.9–3.1%))		
No information available (n (% 95% CI))	20 (3.1% (1.9–4.8%))	15 (2.4% (1.3–3.9%))		
Prehospital physician informed of pre-existing illness			Chi^2^ (2 × 7 table)	<0.0001
No known illness (n (% 95% CI))	335 (52.2% (48.2–56.1%))	291 (46.0% (42.0–49.9%))		
Malignancy (n (% 95% CI))	23 (3.6% (2.3–5.3%))	79 (12.5% (10.0–15.3%))		
Cardiac disease (n (% 95% CI))	114 (17.8% (14.9–20.9%))	60 (9.5% (7.3–12.0%))		
Neurological disease (n (% 95% CI))	31 (4.8% (3.3–6.8%))	38 (6.0% (4.3–8.1%))		
Chronic Obstructive Pulmonary Disease (n (% 95% CI))	50 (7.8% (5.8–10.1%))	58 (9.1% (7.0–11.7%))		
Substance abuse (n (% 95% CI))	32 (5.0% (3.4–7.0%))	20 (3.2% (1.9–4.8%))		
Other (n (% 95% CI))	57 (8.9% (6.8–11.4%))	87 (13.7% (11.2–16.7%))		

### Principal findings

Among the 1275 patients in whom the decision to resuscitate or not was made by the MECU physician, only 62 patients (4.9% (3.7–6.2%) had medical records containing specific ethical or philosophical considerations pertaining to the event. In these 62 patients, a total of 85 individual observations of ethical considerations were made (Table 2).

**Table 2:** Distribution of the ethical issues found in the prehospital discharge summaries. Considerations centered around patients, relatives, or other parties

	Ethical considerations	Total number
1. Patient	Do-not-resuscitate order or note from doctor	38
	Reported wishes and outlook regarding resuscitation	3
	Life expectancy	17
	Quality of life	21
2. Relatives	Emotional states of relatives	2
	Wishes and outlooks regarding resuscitation	4
3. Future patients, medical staff or general public		0
Total number of ethical considerations		85

#### Do-not-resuscitate order

In 36 of the 633 patients not attempted resuscitated (5.9% (4.0–7.8%), a DNR-order was presented to the MECU anaesthesiologist. In two patients (0.3% (0.0–1.1%) of the 642 patients in whom resuscitation was attempted, the MECU physician was aware of a DNR-order when initiating the resuscitation. All DNR-orders were either formulated as written or verbal instruction from the patient, the patients’ general practitioner, the hospital in charge of treatment, or the next-of-kin.

Considerations pertaining to the patient’s quality of life:

- In 21 of patients (3.3% (2.1–5.0%)) in whom natural death was allowed, the main consideration influencing the physician to not initiate treatment was the patients’ expected quality of life after the incident.
- In 17 patients (2.9% (1.6–4.3%)), the physician’s reason to refrain from treatment was the patient’s estimated life expectancy following a hypothetically successful resuscitation attempt.
- In a total of six patients (0.9% (0.3–2.1%)), the reasons for not initiating any resuscitation attempts but to allow natural death to happen was the knowledge of end-of-life wishes or the expectations of the patients or the relatives.

**Fig. 1:**
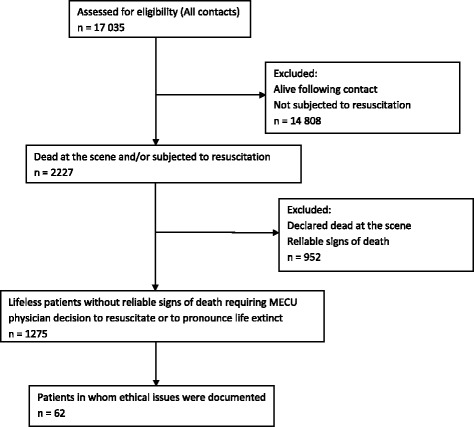
Flowchart

The cases in which ethical considerations were documented included five cases in which treatment had been initiated at the scene despite either obvious signs that treatment would be unsuccessful or even despite an explicit do-not-resuscitate order. In two cases, treatment was initiated despite DNR-orders issued by the general practitioner. These DNR-orders however, were overruled by the patient’s relatives insisting on treatment of the patient. A third case was an infant found dead in her cot. Despite the physician documenting that the treatment was considered obviously futile, the perception that the parents were not ready to accept that their child was dead led the physician to initiate treatment at the scene. In a further two patients, the treatment that had been initiated before the arrival of the MECU physician at the scene led to continuation or escalation of treatment. In one patient, a 90 year old patient with cardiac arrest, the initial treatment resulted in return of spontaneous circulation (ROSC). In the exact moment that ROSC occurred, further information regarding the patient revealed that there was no potential for rehabilitation. As ROSC had been achieved, the physician felt obliged to intubate and ventilate the patient, thus escalating therapy. Likewise, in a fifth patient, resuscitation initiated by a patient’s family led to resuscitative attempts by the MECU physician despite the existence of a DNR-order issued by the patient (Fig. 1, Tables 1 and 2).

Within the material, we found no documentation of additional ethical concerns, for instance concern for the interests of the physician, EMT, nursing staff or chance passers-by (e.g., in car accidents) and issues of prioritisation of resources in the larger perspective of the society.

## Discussion

### An overall evaluation of the documentation can be summed up in five findings

A first finding is that documentation of ethical considerations in the patients’ medical files is exceedingly rare. This is no doubt in many cases just a reflection of the fact that there was no room for ethical decision-making in the circumstances since the medical assessment conclusively established the futility of further life-saving efforts. However, given that decisions regarding patients in whom death is not obvious often involve difficult ethical considerations, it is reasonable to conclude that many actual ethical considerations have gone undocumented.

A second finding pertains to the quality of the existing documentation. In many cases, the description of the ethical considerations was vague or unclear. For instance, when the views of relatives were mentioned it was often not clear whether these were included in the decision as a source of information regarding the wishes of the patients or was part of an argument from the interests of the relatives. In addition, it was difficult to distinguish between ethical and medical reasons for refraining from treatment. For instance, it was often not obvious whether the term ‘futile’ as used in the medical files signified that treatment would not revive the patient or at most lead to a life with a very low functional status (a purely medical assessment) or whether it meant that life would not be worth living even if the patient survived (an ethical assessment). A similar example was the use of the phrase “overall assessment” which often preceded the decision to terminate or not to initiate resuscitative efforts. In these cases it was unclear whether the assessments were purely medical or whether an evaluation of the patient’s expected quality of life after a successful resuscitation was included.

A third finding concerns the content and character of the identified ethical considerations. The ethical considerations found in the material almost exclusively concerned the interests of the patient. The interests most often mentioned were the quality of life and life expectancy, but some medical records also referred to the patient’s previously stated advance directives (as referred to by nursing staff or relatives). These cases must be perceived as a matter of respect for the patient’s autonomy.The material, however, also contained some examples of considerations concerning the interests and emotional states of relatives. For instance, the physician may have described that the relatives were not prepared to accept that further treatment was futile and that the patient was dead.It is both ethically well founded and to be expected that ethical considerations are primarily centered on the patient. Although it has not been documented in our material, it is possible that non-patient centered considerations (costs to society, risks for the personnel, etc.) may have influenced the decision to refrain from treatment.

A fourth finding was that the vast majority of cases in which ethical content was identified were cases where ethical considerations indicated that resuscitative effort might have resulted in an immediate survival albeit with severely reduced quality of life. However, there were two striking exceptions. These were cases where resuscitative efforts were prolonged although from the onset were considered to be futile. The prolonged effort was deemed appropriate out of concern for the relatives present at the scene. In one case, a baby found with sudden infant death was attempted resuscitated and rushed to hospital in consideration of the shock and agony of the parents present at the scene. In another case, resuscitation of a terminally ill patient was initiated despite a medical assessment opposing the resuscitation simply because the relatives were not “ready to accept the death yet”. These cases highlight the potential for ethical conflicts between concern for the patient (e.g., the expressly stated wishes of the patient or the right to posthumous bodily integrity) and concern for the wishes and emotional status of the surviving relatives. This serves further to underscore that ethical decision-making in a prehospital setting is not always trivial or obvious.

A fifth finding concerned the knowledge of the patients’ previous diseases. If the anaesthesiologist at the scene was aware that a patient had been diagnosed with a cardiac disease, this did not seem to restrict the anaesthesiologists in initiating resuscitative efforts. This finding may indicate the awareness of the increasing therapeutic possibilities when treating patients with both heart failure and patients with acute coronary syndromes [9, 10]. However, in patients who had previously been assigned a malignant diagnosis, the prehospital anaesthesiologists were more prone to terminate or refrain from resuscitation. This finding may reflect that withholding the treatment of patients in the terminal stage of a malignant disease may be considered decisions based on beneficence and the principle of non-maleficence (the principle of primum non nocere) by the prehospital anaesthesiologist [3]. Considerations pertaining to whom, what and where may also have played a part in the decision-making. This was relevant both when considering the place of the incident as well as the personnel surrounding the patient when falling ill. Among the group of patients not subjected to resuscitative measures, there a disproportionately larger number of patients were found in nursing homes or in the presence of health care workers. This may reflect a greater need for pre-incident care and as such a lesser likelihood of a good outcome following attempts to resuscitate.

It is highly improbable that all lifeless patients not yet displaying reliable signs of death should be subjected to resuscitative attempts. In at least some cases, respect for the autonomy of a patient ought to prevent the physician from performing resuscitative measures [4]. In other cases, where it is obvious from the onset that survival is impossible, initiating resuscitation can be perceived as an unnecessary and undignified procedure. Indeed, in an older study investigating the conceptions of emergency medical technicians, several EMTs mentioned these aspects of resuscitation and considered that alleviating of stress and pain expressed by the relatives was a more important job [11].

In this study, we have found that in many cases where prehospital critical decision-making considering resuscitation must have taken place, documentation of ethical considerations was exceedingly sparse and often very vague. The documented ethical considerations thus did not correspond to the recommendations in the area [4]. As such, there were never explicit references to considerations relating to the respect for the patient’s autonomy, the principle of beneficence, the principle of non-maleficence, or references to considerations pertaining to justice and avoidance of inequality [4, 12, 13].

In light of our findings, it seems appropriate to discuss whether the current practice regarding documentation of ethical consideration in prehospital emergency care is adequate. We would like to make three observations:

- Firstly, when resuscitative efforts are withheld from the patient, the patient usually dies. Accordingly, the decision to withhold resuscitative therapy is almost always a self-fulfilling prophecy when based on the assumption that a patient cannot be resuscitated. Hence, no evidence of less than optimal decisions is likely to emerge in medical records, i.e., cases where a patient survives despite the absence of resuscitation and has significant benefit from it. This calls for alternative ways of quality-assuring the decisions.
- Secondly, it should be taken into account that there is no easy solution to the potential ethical problems. Merely recommending resuscitation when in doubt in an attempt to always err on the side of caution is not a viable option. Not only can this approach result in patients left with severe handicaps following resuscitation attempts that should never have been initiated, other missions may be left unattended while the MECU is preoccupied with this procedure. Furthermore, emergency transportation of patients undergoing resuscitative attempts, poses a hazard to the MECU crew as well as other people in the streets [14].
- Thirdly, within the hospital, the decision to terminate or to refrain from resuscitative therapy is usually made by a group of physicians and other caretakers following plenary-type consultations, often taking into consideration the expectations and end-of-life wishes of patient and relatives. This in itself is clearly a practice that tends to enhance and assure the quality of the ethical decisions made. The requirement of an explicit formulation of the ethical considerations that comes with the public nature of plenary-type consultation arguably has a positive influence on the quality of the decision-making [15, 16]. This presumed quality-enhancing effect of decision-making is absent in the pre-hospital setting. In some cases, nursing home staff or relatives are at the scene, but more often only one physician and one to three EMTs are present. Formally and legally, however, the authority to decide to either withhold or to terminate resuscitative measures rests with the physician present. A DNR-order can in some cases aid the decision process, but due to the nature of most prehospital deaths, it is exceedingly rare to encounter DNR- orders at the prehospital scene. Thus, the decision to resuscitate or not lies solely with the physician at the scene and must be made based on considerations taking only into account the available limited patient-related information.

**Fig. 2:**
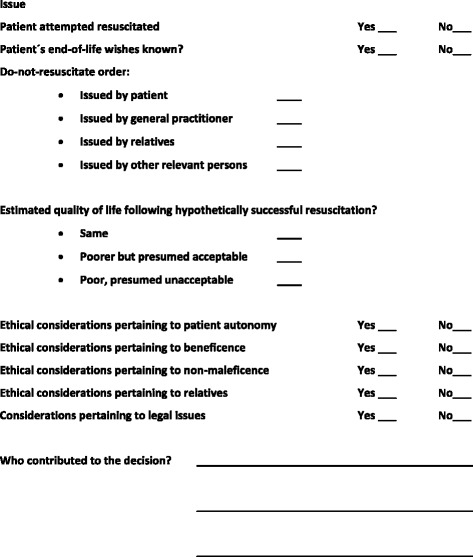
Template for documentation of ethical issues concerning initiation of or refraining from resuscitation

These points, especially the substantial difference in the circumstances of decision-making in prehospital and in-hospital settings and the sparse and vague documentation found in the current practice, appear to call for special measures to secure the quality of ethical decision-making in prehospital settings. An improved practice of documentation based on public guidelines arguably has the potential to offer some substitute for the quality-enhancing effect of collegial consultation. In addition to this, having better documentation is a prerequisite for further studies regarding best practice and can help prevent or dispel myths surrounding end-of-life decisions (e.g., suspicions of unfair treatment of specific groups of people). We propose that a template concerning ethical considerations pertaining to life-and-death decisions be implemented prehospitally (See Fig. 2).

### Strengths and limitations

The strength of the present study is the thorough review of all medical records and databases pertaining to each individual mission. The information registered has been reviewed independently by three authors (SM, CSM, LGB). Also, no patients are lost to follow-up. This is in particular due to executive orders issued by the Danish Ministry of Health stating that a physician is legally required to register any treatment administered to patients [7]. The question of ethical issues surrounding life-and-death decisions is probably universal, and we thus assume that our research question is relevant to other prehospital organisations.

A considerable limitation of our study is that the study addresses patients cared for by one organization only. Our results may thus reflect the documentation culture of this single institution. However, the basic characteristics of the patients and missions are almost identical to those reported in other Danish and Scandinavian studies [17, 18]. Furthermore, with the Scandinavian systems being almost identical, it is possible that the results of this study can prove to be generalizable for Scandinavia [19].

## Conclusion and Recommendations

We believe that there is a need for an improved practice of documenting ethical considerations in pre-hospital life-and-death decisions.When treating patients in whom decisions regarding termination or refraining from resuscitative efforts are relevant, we thus suggest that a compulsory template be implemented in the prehospital medical records describing the basis for any decision being made.In particular, we suggest that guidelines could recommend explicit inclusion in the medical records of the following points:

- ◦ A systematic recording of persons involved in the deliberations (e.g., relatives, nursing staff, or a note from the patient’s doctor).
- ◦ Notes on ethical considerations, including for instance:
  - ◦ Information about the patient’s end-of-life wishes (e.g., a DNR-order or more informal information)
  - ◦ Estimations of quality of life before and after the incident
  - ◦ A summary of other ethical concerns taken into account such as the integrity of the patient and frame of mind of relatives
